# Sphingolipid Long-Chain Base Signaling in Compatible and Non-Compatible Plant–Pathogen Interactions in Arabidopsis

**DOI:** 10.3390/ijms24054384

**Published:** 2023-02-23

**Authors:** Mariana Saucedo-García, Ariadna González-Solís, Priscila Rodríguez-Mejía, Guadalupe Lozano-Rosas, Teresa de Jesús Olivera-Flores, Laura Carmona-Salazar, A. Arturo Guevara-García, Edgar B. Cahoon, Marina Gavilanes-Ruíz

**Affiliations:** 1 Instituto de Ciencias Agropecuarias, Universidad Autónoma del Estado de Hidalgo, Tulancingo 43600, Mexico; 2 Department of Botany and Center for Quantitative Cell Imaging, University of Wisconsin-Madison, Madison, WI 53706, USA; 3 Departamento de Bioquímica, Facultad de Química, Universidad Nacional Autónoma de México, México City 04510, Mexico; 4 Departamento de Biología Celular y Desarrollo, Instituto de Fisiología Celular, Universidad Nacional Autónoma de México, México City 04510, Mexico; 5 Departamento de Biología Molecular de Plantas, Instituto de Biotecnología, Universidad Nacional Autónoma de México, Cuernavaca 62210, Mexico; 6 Center for Plant Science Innovation, Department of Biochemistry, University of Nebraska-Lincoln, Lincoln, NE 68588, USA

**Keywords:** fumonisin B1 (FB1), effector-triggered immunity (ETI), long-chain bases (LCBs), mitogen-activated protein kinase 6 (MPK6), non-compatible interaction, pathogen plant defense, plant disease, programmed cell death (PCD), reactive oxygen species (ROS), sphingolipids

## Abstract

The chemical diversity of sphingolipids in plants allows the assignment of specific roles to special molecular species. These roles include NaCl receptors for glycosylinositolphosphoceramides or second messengers for long-chain bases (LCBs), free or in their acylated forms. Such signaling function has been associated with plant immunity, with an apparent connection to mitogen-activated protein kinase 6 (MPK6) and reactive oxygen species (ROS). This work used in planta assays with mutants and fumonisin B1 (FB1) to generate varying levels of endogenous sphingolipids. This was complemented with in planta pathogenicity tests using virulent and avirulent *Pseudomonas syringae* strains. Our results indicate that the surge of specific free LCBs and ceramides induced by FB1 or an avirulent strain trigger a biphasic ROS production. The first transient phase is partially produced by NADPH oxidase, and the second is sustained and is related to programmed cell death. MPK6 acts downstream of LCB buildup and upstream of late ROS and is required to selectively inhibit the growth of the avirulent but not the virulent strain. Altogether, these results provide evidence that a LCB– MPK6– ROS signaling pathway contributes differentially to the two forms of immunity described in plants, upregulating the defense scheme of a non-compatible interaction.

## 1. Introduction

Because of their natural mechanisms of resistance to infection, plants are not colonized by the majority of potential microbial pathogens. This capacity is due to different defense mechanisms, including the presence of physical barriers, such as the cuticle and cell wall, and the constitutive production of antimicrobial compounds. In addition, sophisticated schemes of pathogen recognition and defense are displayed [1]. Among these, the so-called non-host immunity, a less specific defense response against non-host pathogens, is a potent form of protection with a high durability [2]. In addition, two plant immunity systems have been clearly recognized. They are differentiated by distinct molecular elements, expression kinetics, specificity, effectiveness, and endurance of the elicited response in the host [3,4]. One form of immunity is PAMPs/MAMPs-triggered immunity (PTI) based on the model of recognition of pathogen/microbe associated molecular patterns (PAMPs/MAMPs). In this system, the defense response is produced against pathogen compounds that are conserved throughout a great number of species and therefore is an important, basal, non-specific transient response in the host. PTI is considered the first immunity layer [1]. Another defense scheme is the so-called effector-triggered immunity (ETI), which is based on the gene-to-gene response that corresponds to the highly specific interaction between gene-encoded protein effectors produced by the pathogen and the elicited host resistance proteins (R) gene-encoded as well [5]. When one of the effectors activates the system of a R protein, ETI is initiated and the pathogen growth is limited [6]. ETI is mainly characterized by the robust response against pathogen infection, and it is frequently associated with the hypersensitive response (HR), which involves programmed cell death (PCD). Besides being extremely specific, ETI immune response is powerful and long-lasting [7].

Despite the different recognition and activation mechanisms of PTI and ETI, both classes of immunity converge in the expression of some defense responses such as Ca^2+^ influx, activation of mitogen-activated protein kinases (MAPKs), reactive oxygen species (ROS) production, phytohormone action display and transcriptional reprogramming [8]. MAPKs are signaling components that convert stressor signals into appropriate cell responses. MAPKs function as signaling cascades that require consecutive activation by phosphorylation through a MAPK kinase kinase (MAP3K or MEKK), a MAPK kinase (MAP2K or MKK) and a MAPK (or MPK) to be activated [9]. In *Arabidopsis thaliana* (Arabidopsis), MPK3 and MPK6 activation is transient during PTI but is prolonged during ETI [10,11]. Upon PAMPs/MAMPs perception, the membrane localized NADPH oxidase has been recognized as the source of a ROS burst that takes place in the extracellular space a few minutes after a pathogen exposure [12]. However, these early acting ROS are not related to HR [13], unlike a second massive oxidative emission, which is restricted to non-compatible interactions (the relationship between a plant with an avirulent pathogen that results in an unsuccessful pathogen infection) [14]. Several studies have indicated that ROS generated in the chloroplasts play a key role in the execution of PCD [15,16,17]. ROS accumulation in the plastids is mediated by MAPK cascades, and it causes a dysfunction of the photosynthetic electron transport chain [11,17,18].

*Pseudomonas syringae* pv. *tomato* strain DC3000 (*Pst* DC3000) is a bacterial pathogen of tomato and Arabidopsis and is thus considered a virulent strain that establishes a compatible interaction with the plant. Arabidopsis R protein (RPM1), which recognizes the avrRPM1 effector from *Pst* DC3000 (*avrRPM1*), an avirulent strain, triggers the HR and confers resistance to the pathogen [19]. During this non-compatible interaction between *Pst* DC3000 (*avrRPM1*) and Arabidopsis, endogenous sphingolipid long-chain bases (LCBs) accumulate and precede the onset of HR during ETI, implying that LCBs plays a role in pathogen resistance [20].

Long-chain bases are the metabolic precursors of complex sphingolipids when they are synthesized by the de novo pathway in the endoplasmic reticulum [21]. LCBs are the result of a condensation reaction between serine and palmitoyl-CoA through serine palmitoyltransferase (SPT) [21]. LCBs can vary in chain length and in number and location of hydroxylation and unsaturation sites. In plants, the predominant LCBs are C18 amino alcohols such as the trihydroxy LCB 4-hydroxysphinganine (t18:0) or phytosphingosine (PS) and dihydroxy LCBs as dihydrosphingosine (d18:0) or sphinganine (SN) and 8-sphingenine (d18:1Δ8) [22]. Dihydroxy LCBs, which contain two hydroxyl groups, are converted to trihydroxy LCBs, which possess three hydroxyl groups, by the activity of the sphingoid base C-4 hydroxylase or SBH [23]. LCBs can be acylated by ceramide synthases to form ceramides that provide the hydrophobic backbone of glycosphingolipids. Free and phosphorylated LCBs and ceramides have emerged as signal transducers in plant defense responses against pathogens [24,25,26,27,28] but also in responses to stresses as drought and low temperature [29,30,31,32].

Fumonisin B1, a toxin secreted by *Fusarium verticillioides,* has been a powerful tool in understanding the function of LCBs in defense responses [33,34]. Because of its similar structure to the LCBs, FB1 functions as a competitive inhibitor of selected ceramide synthases [35,36]. FB1 triggers the accumulation of LCBs, their phosphorylated forms (LCBs-P) and ceramides enriched in dihydroxy LCBs in Arabidopsis, which are primarily associated with FB1-induced cell death [23,26,37].

In this study, through the combined use of Arabidopsis mutant lines, FB1 and LCB treatments and the exposure to two strains of *Pseudomonas syringae*, experimental evidence is provided that describes how the crosstalk among LCBs, ROS and MPK6 induces defense reactions in response to an LCB surge and during the non-compatible interaction model.

## 2. Results

### 2.1. Strategy That Provides Integrated Experimental Evidence on the Articulated Participation of LCBs, MPK6 and ROS in Plant Immunity

An in planta strategy combining reverse genetics and a pharmacological approach was used in order to study the roles of LCBs, MPK6 and ROS in innate immunity responses elicited by FB1 or by the avirulent (*avrRPM1*) or the virulent (DC3000) strains of *Pseudomonas syringae* pv*. tomato*. On one hand, the function of specific LCB species and MPK6 was analyzed in the defense expression through reverse genetics using T-DNA insertion null mutant lines for *LCB2a*, *SBH1* or *MPK6* genes (Supplemental Table S1). These Arabidopsis lines are impaired in the expression of genes encoding the LCB2 subunit of SPT, the SBH1 and the MPK6 kinase, respectively. As a result of these mutations, the plants have an imbalance in the content of specific LCB species or lack MPK6 activity (Supplemental Table S1). Notably, the *lcb2a-1* mutant has reductions in LCB biosynthesis, and, conversely, the *sbh1-1* mutant hyper-accumulates dihydroxy LCBs, ceramides, and glycosphingolipids [23,38]. On the other hand, the pharmacological tactic included the use of myriocin, FB1, ketosphiganine (kSN), and the LCBs sphinganine (d18:0, SN) and phytosphingosine (t18:0, PS). Myriocin and FB1 are potent inhibitors of the SPT and ceramide synthase activities, respectively. Myriocin prevents the formation of LCBs and FB1 produces an accumulation of endogenous LCBs [34,39]. To explore the contribution of NADPH oxidase in ROS generation, diphenyleneiodonium (DPI), an inhibitor of this enzyme, was used.

### 2.2. FB1-Elicited Programmed Cell Death Requires Dihydroxy LCBs, MPK6 and ROS

**Figure 1:**
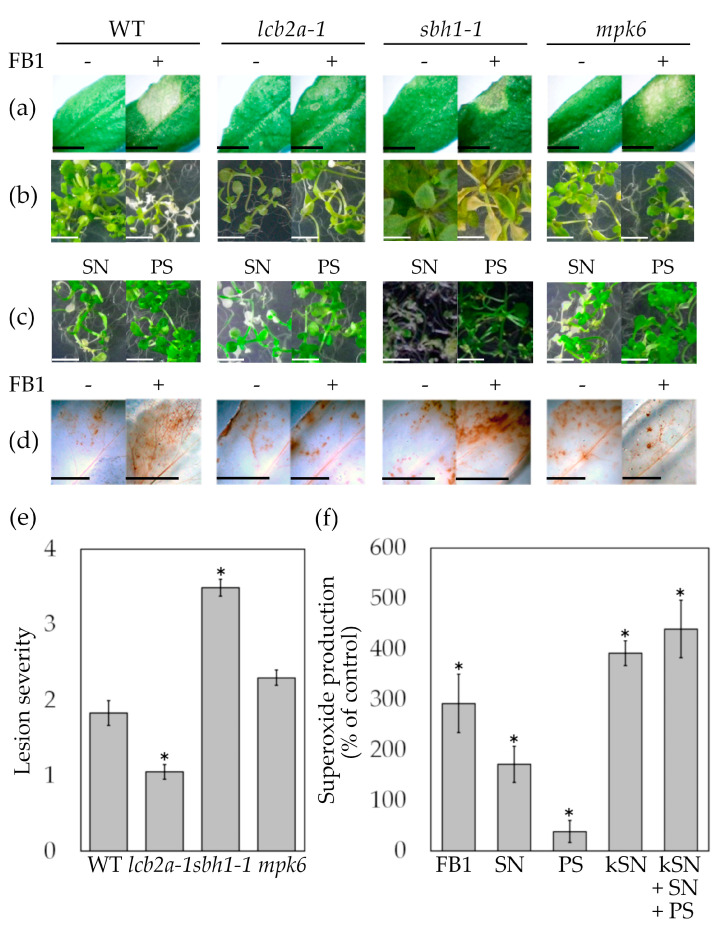
PCD manifestation and ROS production upon FB1 and LCB exposure of Arabidopsis mutants impaired in sphingolipid biosynthesis and MPK6. (**a**) Macroscopic PCD lesions from leaves of adult (10- to 12-week-old plants) or from seedlings (2-week-old) exposed to 10 µM FB1 or 10 mM MgCl_2_ as control (**b**) or 10 µM of SN or PS (**c**). Images were captured at 4 days after treatment for mature plants and 2 days for seedlings. (**d**) In situ generation of H_2_O_2_ in adult plants leaves infiltrated with 10 µM FB1 (+) or 10 mM MgCl_2_ (−) as control. (**e**) Estimation of the lesion induced by FB1 infiltration of the adult leaves (10 to 12-week-old) (see panel 1a) expressed as lesion severity. Lesions were assessed at 4 days after treatment. Data are the mean ± SE of 3 independent experiments. (**f**) Production of formazan in the medium from an Arabidopsis wild-type protoplasts preparation treated with FB1, the LCBs SN, PS or their precursor ketosphinganine (kSN) after 1 h. Data are mean ± SE of 3 independent experiments. Each treatment was compared to the 100% formazan production of the control. Scale bar corresponds to 0.5 cm in all cases. Asterisks indicate significant difference *t* statistically compared to the control (*p ≤* 0.05).

Long-chain bases and MPK6 are important positive regulators of PCD elicited by FB1 in Arabidopsis seedlings [26]. The response of mature wild-type and *lcb2a-1*, *sbh1-1* and *mpk6* plants to the FB1 infiltration was explored in leaves from 10- to 12-week-old plants (Figure 1a). In contrast to the damage observed in the wild-type plants by FB1 infiltration, lesions were reduced in the *lcb2a-1* mutant, *mkp6* showed a similar lesion severity to wild-type, and the *sbh1-1* line showed enhanced lesions at 4 days after FB1 treatment (Figure 1a,e).

Such findings in adult plants were reproduced in the seedlings from the same Arabidopsis lines (Figure 1b), where *lcb2a-1* and *mpk6* lines were less susceptible to the FB1 exposure, as was previously reported [26]. In this study, the complementation effect of the exogenously added LCBs into the mutants affected in the content of endogenous LCBs was tested. Thus, all genotypes were exposed to SN or PS (Figure 1c). As it can be observed, the four genotypes treated with SN showed clear signs of PCD and no effect with PS addition. In the case of the *sbh1-1* mutant, the addition of SN exacerbated the effect of the endogenous excess of this same LCB, which promotes PCD; this was not the case of PS, which complemented the decrease in this endogenous trihydroxylated LCB.

Altogether, these results validated the use of both developmental stages of these Arabidopsis genotypes as experimental systems to study PCD. Moreover, these results using both exogenous LCBs and FB1 as a promotor of endogenous LCB accumulation indicated that the dihydroxylated LCBs are inducers of cell death.

Programmed cell death elicitation by FB1 requires ROS accumulation [25]. To address whether *lcb2a-1*, *sbh1-1* and *mpk6* mutants had changes in ROS levels upon FB1 infiltration, ROS accumulation was monitored in situ by DAB (3,3′-diaminobenzidine) staining in mature leaves after 3 days of treatment. As shown in Figure 1d, DAB-stained areas were increased in the leaves from wild-type and *sbh1-1* plants. Interestingly, *lcb2a-1* and *mpk6* mutants, which showed tissue necrosis at a minor extent (Figure 1a), were unable to accumulate H_2_O_2_ as well. Taken together, these results indicated that accumulation of dihydroxy LCBs and ROS as well as the MPK6 activity were needed for PCD triggered by FB1 in planta.

It is well known that chloroplasts are a vast source of ROS in the FB1-induced PCD [18,40]; hence, this study explored the contribution of apoplastic ROS formation. For these experiments, protoplasts isolated from Arabidopsis wild-type plants were stained with tetrazolium dye (XTT) (Figure 1f). In the presence of O_2_^.−^, XTT is reduced to a soluble formazan, which is measured by spectrophotometry. Protoplasts treated with FB1 or SN produced a significant reduction of XTT (Figure 1f). Additionally, a substantial increase in the dye reduction was observed in response to ketosphinganine (kSN), a precursor of all de novo formed LCBs, either when it was applied alone or in combination with both LCBs. In contrast, protoplasts treated with PS were unable to reduce XTT in the evaluated conditions. These results indicated that ROS production is mainly due to dihydroxy LCBs and that this is coincident with the contribution of these LCBs to PCD. Addition of DPI was used to discern the contribution of NADPH oxidase to the formazan production detected in Figure 1f. The XTT reduction induced by FB1 diminished 18.5% in the presence of DPI (Supplemental Table S2), revealing that NADPH oxidase was partially contributing to an early generation of the intracellular ROS induced by the FB1, as was previously reported by Saucedo-García et al. and Lachaud et al. [26,41].

### 2.3. Resistance against an Avirulent Strain of Pseudomonas syringae Is Mediated by LCBs and Involves MPK6

**Figure 2:**
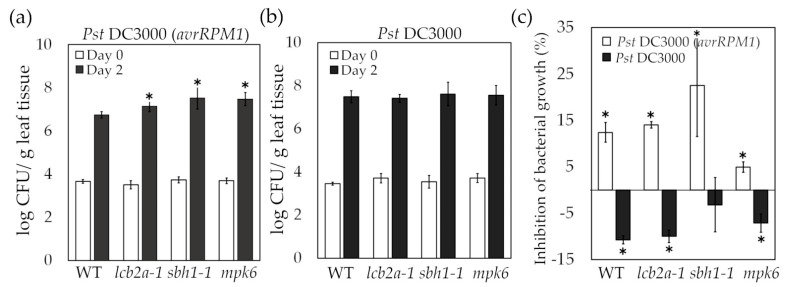
Growth of avirulent *Pseudomonas syringae* DC3000 (*avrRPM1*), or virulent *Pseudomonas syringae* (DC3000) strains in Arabidopsis mutants impaired in sphingolipid biosynthesis and MPK6. Determination of bacterial growth after spray-inoculation of seedlings from the indicated genotypes with 10^7^ CFU/mL of avirulent *Pst* DC3000 (*avrRPM1*) (**a**) or virulent *Pst* DC3000 strains (**b**). (**c**) Effect of FB1 treatment prior to inoculation with *Pst* DC3000 (*avrRPM1*) or virulent *Pst* DC3000 strains on bacteria growth in seedlings. Seedlings were sprayed with 10 μM FB1 and after 12 h were sprayed with 10^7^ CFU/mL of *Pst* DC3000 (*avrRPM1*) or *Pst* DC3000. Bacterial growth was determined at 2 dpi. Percentage was calculated taking 100% as the bacterial growth determined in the absence of FB1 for every genotype at the same time. Values are expressed as the means ± SD from two independent experiments, each with three replicates. The two-tailed Student’s *t*-test was used for statistical analyses. Asterisks indicate significant differences compared to wild-type (**a**,**b**) or without FB1 (**c**) at *p ≤* 0.05.

Similar to the treatment with FB1, infection of Arabidopsis wild-type seedlings with the avirulent *Pst* (*avrRPM1*) or the virulent *Pst* (DC3000) strains also evoked LCB accumulation [20]. While the avirulent interaction leads to a long-lasting elevation of PS levels, the increase in this LCB is transient in the virulent interaction (Supplemental Table S1) [20]. To examine whether the dihydroxy or trihydroxy LCBs and MPK6 were required to the immunity towards *Pst* DC3000 (*avrRPM1*), the bacterial growth was monitored *in planta* (Figure 2a). The *Pst* DC3000 (*avrRPM1*) titers inoculated at day 0 were the same in the different mutants and the wild-type seedlings, as proved by the quantitation of the bacterial population (empty bars). However, the growth of the avirulent strain increased 2.4, 6.08 and 5.41-fold in the *lcb2a-1, sbh1-1* and *mpk6* mutants at 2 days post-inoculation (dpi), respectively, as compared to the wild-type (notice the logarithmic scale). In the case of the virulent strain *Pst* DC3000, the initial bacterial titers inoculated to the wild-type and the three mutants were similar among them as well (empty bars) and comparable to the *Pst* DC3000 (*avrRPM1*) inoculated doses (Figure 2b). However, the increase in bacterial titers of *Pst* DC3000 observed at 2 dpi were statistically equivalent in all genotypes. Overall, this suggested that dihydroxy and trihydroxy LCBs and MPK6 are important regulators of the immune response unchained only by the avirulent strain.

Because FB1 promotes the increase in free LCB levels by inhibiting the activity of selected ceramide synthases, the possibility that the addition of 10 µM FB1 to seedlings 12 h prior to bacterial infection could promote bacterial resistance was evaluated. As shown in Figure 2c, the pre-treatment of the seedlings with FB1 inhibited the growth of *Pst* DC3000 (*avrRPM1*), but not of *Pst* DC3000 in all evaluated genotypes. This revealed that sphingolipid precursors were clearly and differentially associated with the immunity scheme elicited by the *Pst* DC3000 (*avrRPM1*). Furthermore, the results showed that MPK6 activity was required to successfully invoke the immunity response and that this kinase activity was downstream the LCBs build-up. FB1 had no effect on bacterial growth, as it is shown by the bacterial growth in medium supplemented with FB1 (Supplemental Figure S1).

### 2.4. LCBs Are Involved in ROS Accumulation Induced by the Avirulent Pseudomonas syringae Strain

**Figure 3:**
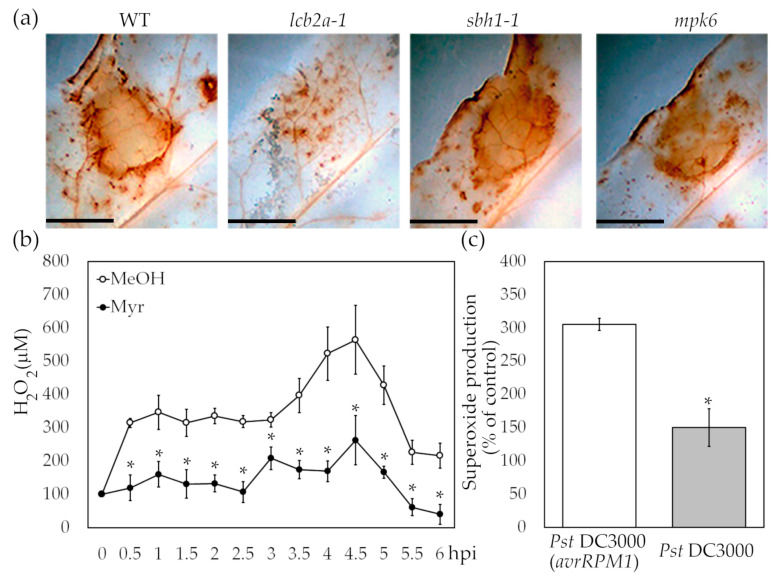
ROS generation at different stages of *Pst* DC3000 (*avrRPM1*) infection. (**a**) In situ detection of H_2_O_2_ in leaves from Arabidopsis wild-type, *lcb2a*-1, *sbh1-1* and *mpk6* adult plants infiltrated with 10^7^ CFU/mL *Pst* DC3000 (*avrRPM1*). Images were captured at 3 dpi. A representative image from each condition is shown in panel (**a**), where independent experiments were replicated 3 times with a total of 6 plants and 12 leaves for each genotype and treatment. (**b**) Accumulation of H_2_O_2_ in the medium from Arabidopsis wild-type cell suspensions upon exposure to 10^7^ CFU/mL *Pst* DC3000 (*avrRPM1*). Measurements were performed in the presence of a methanol dissolved myriocin solution or the dissolvent. (**c**) Formation of O_2_^.−^radical in Arabidopsis protoplasts upon addition of avirulent or virulent *Pst* strains at a concentration of 10^7^ CFU/mL. Measurements were performed after 1 h. See Materials and Methods for details. The results show the means of percentage of the total amount produced in control protoplasts ± SD from three independent experiments. Scale bar corresponds to 0.5 cm. The two-tailed Student’s *t*-test was used for statistical analyses. Asterisks indicate significant differences compared to the solvent used in the myriocin solution (**b**) or to the avirulent strain (**c**) at *p ≤* 0.05.

It is known that ROS have a vital role in plant immunity [42]. To assess the importance of LCBs and MPK6 on ROS production during the resistance to *Pst* DC3000 (*avrRPM1*), the mutant lines were challenged with bacteria suspensions. We found that upon infection, the intensity of the stained area due to formation of H_2_O_2_ in situ was slightly lower in leaves from *mpk6* and particularly low in the *lcb2a-1* mutant compared to wild-type and *sbh1-1* plants (Figure 3a); this indicated that MPK6 and dihydroxy LCBs upregulate ROS accumulation elicited by the avirulent *Pst* strain.

To support the latter finding, myriocin was used in wild-type protoplasts to block SPT activity and thus inhibiting LCB synthesis. Figure 3b shows that protoplasts treated with myriocin and then exposed to *Pst* DC3000 (*avrRPM1*) produced approximately 18–64% less H_2_O_2_ as compared to the control in a time-course from 0.5 h to 6 h. This demonstrates that LCBs are upstream of ROS production in the signaling pathway.

As it was previously shown, infection with *Pst* DC3000 (*avrRPM1*) produced accumulation of superoxide O_2_^.−^ inside Arabidopsis chloroplasts [43]. However, it has also been reported that NADPH oxidase produces an oxidative burst upon an avirulent pathogen attack [44]. Thus, the superoxide production in Arabidopsis protoplasts after exposure to the avirulent or virulent *Pst* strains was determined. As observed in Figure 3c, when the virulent *Pst* strain was added, the protoplasts produced about 50% of the O_2_^.−^ accumulated after exposure to the avirulent strain *Pst*. Additionally, it was found that about 57% of the O_2_^.−^ concentration measured in protoplasts inoculated with *Pst* DC3000 (*avrRPM1*) was caused by NADPH oxidase activity since the use of DPI significantly reduced the formazan content in Arabidopsis protoplasts (Supplemental Table S3), revealing that there are different sources of ROS production with specific allocation, temporality, and intensity in order to confront the bacterial infection.

## 3. Discussion

Fumonisin B1 is an excellent tool to understand the role of sphingolipids on plant signaling, in particular on PCD [34]. FB1 inhibits the activities of ceramide synthases in eukaryotic cells [21,35,36,38,45,46]. The rate of accumulation of trihydroxy LCB species is differentially affected by FB1 as well, since while SN levels increase 619-fold, PS rises 113-fold at 3 days after FB1 addition to Arabidopsis seedlings [26]. Furthermore, the fact that Arabidopsis seedlings showed cell death in a medium supplemented with exogenous SN but not in the presence of PS ([26], this work), strongly suggests that dihydroxy LCBs, free or acylated in ceramide forms are involved in cell death induced by FB1 [36], which is coincident with the ceramides accumulation that contribute to immunity [47]. In the present work, differential sensitivity of *lcb2a-1* and *sbh1-1* lines to FB1 provided evidence to support this hypothesis. These results showed, firstly, that de novo synthesized LCBs are required for FB1-induced cell death and, secondly, that dihydroxy LCBs, free or acylated, are necessary for the successful induction of cell death by FB1.

**Figure 4:**
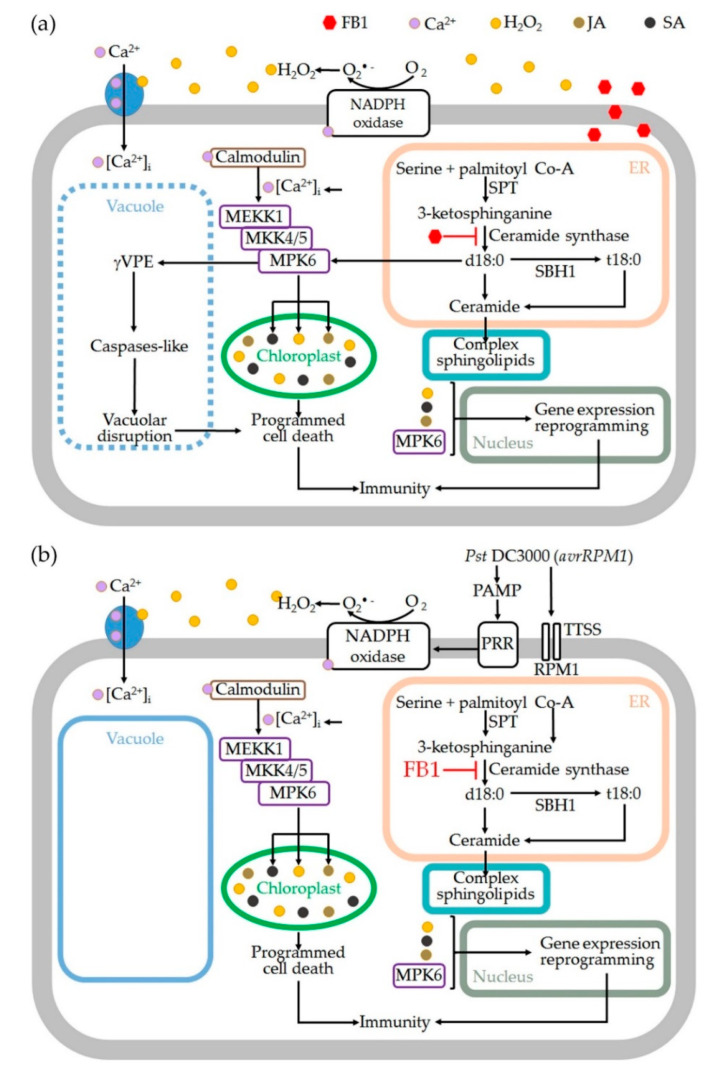
Schematic model of the action of the LCB surge induced by FB1 or by *Pst* DC3000 (*avrRPM1*), an avirulent strain. A rise of LCBs, mainly SN and PS, is produced upon addition of FB1 (**a**) or *Pst* DC3000 (*avrRPM1*) (**b**). This unchains an early oxidative burst by NADPH oxidase. The LCB rise promotes MPK6 activation. Downstream this kinase, an activation of the γVPE, produces vacuolar disruption and, in the chloroplast, a massive ROS production which leads to an irreversible PCD. Jasmonate, ethylene and salicylic-acid-defense-mediated pathways are also activated by MPK6 upon FB1 administration. γVPE, vacuolar processing enzyme; JA, jasmonic acid; PAMP, pathogen-associated molecular patterns; PRR pattern recognition receptors; SA, salicylic acid; TTSS: type III secretion system. PAMP, PRR and TTSS are identified elements expressed under the effector-triggered immunity (ETI).

It is known that signaling through MAPK cascades is also involved in the FB1-PCD elicitation. In *mpk6*, but not in *mpk3*, FB1- or SN-triggered cell death of Arabidopsis seedlings was attenuated [26]. The PCD induced by heat shock that involves the vacuolar processing enzyme (γVPE) requires activation of MPK6 by a post-translational mechanism that promotes caspase-3-like activation leading to vacuolar membrane disruption [48]. Therefore, the increase in γVPE (caspase-1 like) activity triggered by FB1 [49], conducting to vacuolar disruption and cell death in Arabidopsis, is likely to be due to the increase in MPK6 activity (Figure 1 and Figure 4).

Downstream effects of MAPK cascades depend on intensity and temporality of MAPK activity [17,50]. The prolonged activation of MPK3/6 has been associated with HR-like cell death and to an over-accumulation of ROS in chloroplasts in a light-dependent manner [11,51]. This is similar to the observed increased ROS levels in chloroplasts from bean and Arabidopsis leaves treated with FB1 [17,40]. Structural and functional disruption of chloroplasts are common features observed in Arabidopsis cells from leaves treated with FB1 [40]. This disruption is dependent on ROS derived from the chloroplasts since the use of antioxidants or scavengers prevents it [40]. By surveying ROS production associated with gene loss-of-function mutations, the present work illustrates that de novo synthesized LCBs, in particular dihydroxy LCBs and MPK6 along the same signaling wire, were responsible for the upregulation of ROS production and the subsequent execution of PCD triggered by FB1 (Figure 1 and Figure 4).

Besides chloroplasts, there are other sites of ROS production such as the apoplast, mitochondria and peroxisomes [52]. The apoplastic ROS are mainly produced by plasma membrane localized NADPH oxidases (respiratory burst oxidase homologs, RBOHs), cell wall peroxidases and amine oxidases [53]. In the present study, it was found that the production of superoxide anions in the first hour of FB1 or SN treatment of Arabidopsis protoplasts was partially inhibited by DPI. These data suggest that FB1-induced LCB accumulation results in ROS production at a certain extent mediated by NADPH oxidase activity. This early production of superoxide anion coincides with measurements upon 5 μM FB1 addition to Arabidopsis cell cultures [54] and with the ROS produced by exogenous LCBs in Arabidopsis leaf discs [55].

Like FB1, infection with avirulent and virulent strains of *Pst* also evokes a rapid increase in LCBs, mainly of trihydroxy LCBs [20]. In this regard, it has been reported that infection of *Nicotiana benthamiana* with the non-host *Pseudomonas cichorii* induces a strong expression of *LCB2* and that, by silencing the *LCB1* gene, the growth of *P. cichorii* has a drastic increase [56]. In the current study, it was found that loss of function of both *LCB2a,* and particularly, *SBH1* genes severely impaired ETI. Recent studies have shown that PS inhibits bacterial and fungal pathogens growth, alleviating disease symptoms of co-infiltrated virulent and avirulent strains of *Pst* into Arabidopsis leaves [57]. However, this bacteriostatic action of the added LCB was associated with a direct toxic effect on bacteria viability rather to a natural plant defense mechanism. In a similar manner, the co-infiltration of SN with *Pst* DC3000 (*avrRPM1*) in Arabidopsis decreased disease symptoms and electrolyte leakage between 4 and 20 h post infection [26].

Due to the vast evidence about FB1-induced defense responses [25,26,40,49,51,54,58,59], here it was studied whether LCBs increased by FB1 treatment in planta could improve immunity. Induction of this endogenous accumulation of LCBs using FB1, effectively led to a stronger defense towards an avirulent strain in wild-type, *lcb2a-1* and *sbh1-1* plants. Although *lcb2a-1* increased LCBs levels to a lower extent as compared to wild-type 12 h after FB1 treatment [26], the accumulated LCB amount seemed to be enough to affect the bacterial growth of the avirulent strain. In addition, the increase in endogenous dihydroxy LCBs promoted by FB1 in the *sbh1-1* mutant could cause the reduction of the avirulent strain growth during infection. This finding implies that the very early surge of PS showed upon the *Pst* virulent strain infection [20] is unable to elicit a sustained and effective defense response in Arabidopsis, which is consistent with the absence of protection in a compatible interaction. Taken together, these results indicate that de novo synthesized LCBs promote plant immunity triggered by a non-compatible interaction and not by a compatible one and position the LCBs with a very important role in the immunity schemes in plants.

MAPK cascades are fundamental components of the plant innate immune system. The long-lasting activation of MPK3/MPK6 has been associated with ETI [9,11,16,17]. In the present study, it was found that *mpk6* mutant sustained a robust bacterial growth of the *Pst* avirulent strain and a minor sensitivity to the effect of LCBs accumulated by FB1 addition. These results provide evidence on the importance of MPK6 to restrict bacterial growth, reducing pathogenicity during Arabidopsis infection, and reveal the MPK6 action downstream of LCB signaling during ETI.

ROS have a crucial role in plant defense and signaling pathways and also have a direct toxicity against pathogens [60]. NADPH oxidase, in particular RBOHD, is required for most of the ROS signaling species produced in Arabidopsis after inoculation with *Pst* DC3000 (*avrRPM1*) during the very first hours [44]. However, chloroplasts also contribute to a larger ROS production in later stages of the interaction of *Pst* DC3000 (*avrRPM1*) with Arabidopsis or bean plants [11,17,43]; this effect seems to be associated with a destructive effect of ROS on the chloroplast in order to make the cell death irreversible [17]. The role of LCBs in triggering an oxidative burst in the non-compatible interaction Arabidopsis-*Pst* DC3000 (*avrRPM1*) was evaluated in this study through assays that examined the pathogen growth. The results indicated that ROS production induced by the avirulent strain required LCBs. First, *lcb2a-1* showed lower ROS accumulation in situ than wild type. Second, *sbh1-1* (with higher SN levels) showed greater ROS accumulation after *Pst* DC3000 (*avrRPM1*) infection. Third, the co-treatment of cell cultures with myriocin and the *Pst* avirulent strain diminished H_2_O_2_ accumulation over time. Fourth, protoplasts treated with *Pst* DC3000 (*avrRPM1*) produced ROS partially generated by NADPH oxidase during the first hour of infection. Together, these results suggest that the non-compatible interaction between Arabidopsis and *Pst* DC3000 (*avrRPM1*) requires the NADPH oxidase activity in the first hours of infection, and later needs chloroplasts to accumulate ROS to trigger an ETI in which an endogenous surge of LCBs is essential.

Since the prolonged activation of MPK3 and MPK6 has been related to the inhibition of photosynthesis at multiple levels and also with the generation of chloroplastic ROS [11], we hypothesised that the immunity triggered by *Pst* DC3000 (*avrRPM1*) involves MPK6 to induce an apparently sustained but diminished ROS accumulation (Figure 3 and Figure 4) as the *mpk6* line showed an attenuated ROS production.

Collectively, our results and those compiled from the literature place the differential participation of the LCB surge linked to MPK6 and ROS in two PCD schemes as a central feature in plant immunity (Figure 4).

## 4. Materials and Methods

### 4.1. Special Reagents

Cellulase Onozuka RS, diphenileniodonium (DPI), fumonisin B1 (FB1), macerozymeR-10 and myriocin were obtained from Sigma-Aldrich, (St. Louis, MO, USA). Long-chain bases SN and PS were purchased from Avanti Polar Lipids, Inc. (Alabaster, AL, USA). SilwetL-77 was obtained from Chemtura Corporation México, S de RL de CV (Mexico City, Mexico).

### 4.2. Biological Materials

The bacterial strains used were the avirulent and virulent strains *Pseudomonas syringae* pv. *tomato* DC3000 *avrRPM1* (*Pst* DC3000 *avrRPM1*) and *Pseudomonas syringae* DC3000 (*Pst* DC3000), respectively.

The *Arabidopsis thaliana* Col-0 genotypes used in this study were wild-type and mutant lines *lcb2a-1, sbh1-1* and *mpk6*, which were previously characterized (Supplemental Table S1). They were utilized as seedlings (2-week-old) or as adult plants (10- to 12-week-old).

### 4.3. Arabidopsis Growth

For experiments with seedlings, seeds were grown in Gamborg B-5, 1% agar and 1% sucrose medium on rounded Petri dishes horizontally positioned at 22 °C under a photoperiod of 16 h light and 8 h dark. They were exposed to FBI or bacterial strains as indicated below.

For experiments with adult plants, seeds were grown in a mixture of (Mix 4 Aggregate Plus, Sunshine, Sun Gro Horticulture, Canada Ltd. (Manitoba, MB, Canada), vermiculite, Premium Grade, Sunshine, Sun Gro Horticulture; Canada Ltd.; and agrolite, Dica Mex, Dicalite de México S.A. de C.V. (Edo. de México, México) in 3:1:1, *v*:*v*:*v*, respectively), watered on alternate days with water and Hoagland solution and grown at 22 °C under a photoperiod of 16 h light and 8 h dark. Ten- to twelve-week-old plants were exposed to the different treatments as described below.

### 4.4. Callus Obtention

Arabidopsis Col-0 wild-type seeds were surface sterilized for 15 min in 10% sodium hipoclorite and 0.05% (*v*/*v*) Triton X-100 and then washed four times with sterilized water. Seeds were dried on an autoclaved Whatman paper for at least 10 min. Seeds were subsequently plated on Murashige and Skoog medium containing 3% (*w*/*v*) sucrose, 0.05% MES [p:v], pH 5.7 and constantly stirred at 150 rpm to be germinated at 22 °C under a 16 h light/8 h dark photoperiod.

Cotyledon pieces from Arabidopsis wild-type 2-week-old seedlings grown in sterile conditions were transferred onto callus induction solid medium (Murashige and Skoog medium, as above but supplemented with 5.37 μM naphthaleneacetic acid, 0.46 μM kinetin, vitamin B5, 0.3% (*w*/*v*) gellan gum, and 5 μg/mL kanamycin). Callus fractions were transferred onto fresh medium every 2 weeks.

### 4.5. Cell Suspension Cultures

They were obtained transferring 3 g of well-developed calli into 30 mL of liquid MS medium containing the same concentration of sucrose and plant hormones as the solid medium, supplemented with 5 μg/mL kanamycin. Calli were gently stirred and maintained at 22 °C with 16 h light/8 h dark photoperiod and were subcultured every week. Grown cell aggregates obtained after several weeks were filtered to obtain small cell cumuli, which were allowed to descend to the bottom, the old medium was discarded and 10 mL of medium containing this material was transferred to a new flask containing 30 mL of fresh medium, which was replaced every 2 weeks. Once that cell density was adequate, protoplasts were isolated [61].

### 4.6. Protoplast Preparation and Determination of Superoxide Anion

Protoplasts were obtained from 7-day-old cell suspension cultures from Arabidopsis wild-type. Cell walls were digested with 1% (*w*/*v*) cellulose Onozuka RS and 0.2% (*w*/*v*) with macerozyme R-10 in 0.5 M mannitol, 5 mM KCl and 2 mM CaCl_2_. Mixture was gently shaken at 40 rpm for 15 h at 22 °C under dark conditions. After incubation, the suspension was centrifuged at 1000 rpm at 22 °C for 2 min under dark conditions. The fraction over the pellet was recovered and filtered through a nylon mesh of 0.62 µM. The filtrate was collected and centrifuged at 1000 rpm at room temperature. Protoplasts were collected from the surface and counted.

### 4.7. FB1 Treatments

A 10 μM FB1 solution was prepared in sterile 10 mM MgCl_2_ and applied in the treatments. Arabidopsis seedlings grown in Gamborg’s medium in Petri dishes were exposed to FB1 by spraying 0.6 mL of the FB1 solution to Petri dishes of 4.5 cm diameter or 1.0 mL to petri dishes of 8 cm diameter. Controls were exposed to 10 mM MgCl_2_. They were kept under continuous light at 22 °C and phenotypical responses were recorded in photographs. Every biological replicate, from a total of 3, consisted of 6 petri dishes (3.5 cm diameter) per genotype per treatment.

Arabidopsis adult plants were exposed to FB1 by infiltration of 20 μL of the FB1 solution on the abaxial right side of the leaves at one point in the middle region using a 1.0 mL needleless syringe. Phenotypical responses were recorded in photographs. Every biological replicate, from a total of 3, consisted of at least of 5–6 treated leaves from independent plants, and 3 independent experiments were performed with a total of 9 plants and 18 leaves per genotype per treatment. 

### 4.8. LCB Treatments

A volume of 0.7 mL containing 10 µM SN or PS dissolved in 0.04% (*v*/*v*) Silwet L-77 was sprayed onto 3-week-old Arabidopsis seedlings grown in Petri dishes (3.5 cm diameter). Control seedlings were sprayed with the Silwet solution. Seedlings were maintained under continuous light at 22 °C during the treatment.

### 4.9. Bacterial Treatments

*Pseudomonas syringae* pv. *tomato* DC3000 *avrRPM1* (*Pst* DC3000 *avrRPM1*) and *Pseudomonas syringae* DC3000 (*Pst* DC3000) strains stored in 25% glycerol solution at −80 °C were grown in King’s B medium containing 50 µg/mL rifampicin (for *Pst* DC3000) or 20 µg/mL tetracycline and 50 µg/mL rifampicin (for *Pst* DC3000 *avrRPM1*) at 29 °C under dark conditions. Samples were taken from these strains and fresh cultures were obtained to prepare bacterial suspensions in 10 mM MgCl_2_ with an OD_600 nm_ of 0.1 O.D.U., equivalent to 1 × 10^8^ CFU. A 1 × 10^7^ CFU dilution was prepared and sprayed to the seedlings. They were kept under continuous light at 22 °C and phenotypical responses were recorded in photographs or quantitation of bacterial growth was performed. Every biological replicate, from a total of 3, consisted of at least 2 plates of control and treated seedlings.

### 4.10. Quantitation of Bacterial Growth in Planta

The progress of bacterial growth in planta was determined as described in Yang et al. [62]. Briefly, 0.1 or 0.25 g portions of seedlings grown in Petri dishes were washed in 70% ethanol solution for 30 s, paper blotted and then washed in sterile water for 30 s. Then, they were ground in small plastic bags with 300 μL of 10 mM MgCl_2_ by external pressure with a mortar pestle. Serial dilutions were performed from these homogenates, plated in solid B King medium as described above and incubated at 29 °C for 36 to 48 h, and the bacterial growth was estimated. FB1 had no effect in the growth of *Pseudomonas syringae* pv. *tomato* DC3000 *avrRPM1* (*Pst* DC3000 *avrRPM1*) or *Pseudomonas syringae* DC3000 (*Pst* DC3000) (Supplemental Figure S2).

### 4.11. Determination of In Situ H_2_O_2_ Generation

FB1-infiltrated leaves were stained with 3,3-diaminobenzidine (DAB) solution (1 mg/mL, pH 3.8) under white light for 2 h and then decolored in 96 % ethanol [63]. Then, leaves were hydrated in glycerol/H_2_O/acetic acid (70/20/10, *v*/*v*/*v*) and photographed with a Motic 100 camera coupled to a Zeigen microscope. The experiments were independently repeated at least 3 times with a total of 6 plants and 12 leaves per genotype per treatment.

### 4.12. Determination of H_2_O_2_ in Solution

Hydrogen peroxide (H_2_O_2_) was measured in the medium of the cell suspensions using the procedure described in Sagisaka et al. [64]. The suspension cells were treated with methanol as control or 100 nM myriocin and incubated at 22 °C under light. After incubation, 1000 μL of extracellular fluid was withdrawn and then incubated in a phosphate buffer for 4 min (30 mM MES, 120 mM NaCl, 1.2 mM KH_2_PO_4_, 1 mM NaN_3_, 100 μM FAD, 0.25 mM NADPH). After incubation, 400 μL of 1.5 M trichloroacetic acid (TCA) was added and the fluid was centrifuged at 10,000 rpm at 4 °C for 10 min. Then, 900 μL of supernatant was withdrawn and supplemented with 200 μL of 10 mM (NH_4_)_2_Fe(SO_4_)_2_ 6H_2_O and 100 μL of 2.5 M KSCN. The absorbance was recorded at 480 nm using a T60 UV/VIS, spectrophotometer (PG Instruments Limited, Leicestershire LE17 5BH, UK). The standard curve was prepared using 0.88 μM H_2_O_2_. Measurements of H_2_O_2_ concentration were done in three independent experiments that included three biological replicates.

### 4.13. Determination of the Extent of Lesions Induced by FB1

This was estimated by visual comparison of the appearance of the lesion to a scale established according to a series of lesions induced by FB1 infiltration that showed increased intensity (Supplemental Figure S2). The severity of the damage was determined by estimation according to the following scale, where n = 0 (no damage) to n = 5 (severe damage). The lesion severity (LS) was calculated according to the equation LS = Σ (leaves × n)/total number of leaves. For Figure 1a,e, the progress of the lesion induced by FB1 was assessed at 4 d post-infiltration.

### 4.14. Determination of O_2_^.−^Radical

A solution of XTT (sodium;4-methoxy-5-[3-(2-methoxy-4-nitro-5-sulfonatophenyl)-5-(phenylcarbamoyl) tetrazol-3-ium-2-yl]-2-nitrobenzenesulfonate) at a final concentration of 0.2 mM was added to a preparation of Arabidopsis protoplasts at 1 × 10^7^ CFU/mL. After 15 min of incubation, bacterial suspensions were added at the indicated times. Diphenyleneiodonium (DPI), a NADPH oxidase activity inhibitor, was added at 50 μM final concentration after the XTT incubation with the protoplasts, and 10 or 100 μM sphinganine (SN), a dihydroxylated LCB and 10 μM FB1 were added after the 15 min incubation as well. Then, 2.0 mL aliquots were withdrawn at different times and vacuum filtrated, and absorbance was measured at 470 nm. Three experiments with independent protoplast preparations were performed for every condition.

### 4.15. Statistical Analysis

Data shown are mean values with SE from three independent experiments with three individual replicates (unless indicated otherwise). Significance levels were tested by a two-tailed Student’s *t*-test with an α of 0.05.

## Data Availability

Not applicable.

## Supplementary Materials

The following supporting information can be downloaded at: https://www.mdpi.com/article/10.3390/ijms24054384/s1. References [65,66] are cited in the supplementary materials.
