# Supplementary material for: Sphingolipid Long-Chain Base Signaling in Compatible and Non-Compatible Plant–Pathogen Interactions in Arabidopsis
Source: Int J Mol Sci. 2023 Feb 23;24(5):4384. doi: 10.3390/ijms24054384 (PMC10002605; doi:10.3390/ijms24054384)
Supplement: Supplementary file 1 [file ijms-24-04384-s001.zip › Supplemental Figure S1.pdf]

Supplemental Figure S1

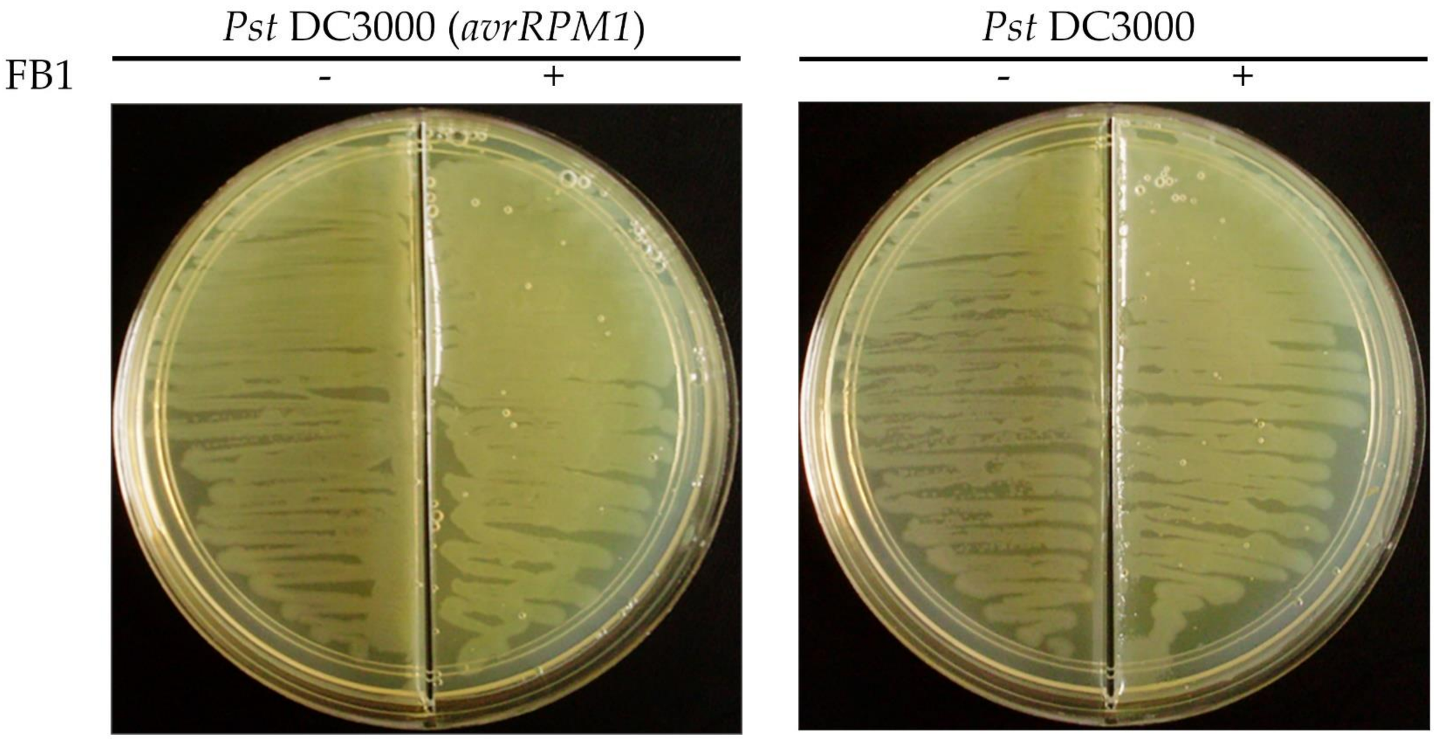

Supplemental Figure S1. FB1 has no effect on *Pseudomonas syringae* pv. *tomato (Pst)* growth. The avirulent strain DC3000 avrRPM1 (*Pst* DC3000 *avrRPM1*) or the virulent strain DC3000 (*Pst* DC3000) were used along this work. Bacteria were strained in B King´s medium supplemented with rifampicin/tetracycline (for *Pst* DC3000 *avrRPM1*) or rifampicin (for *Pst* DC3000) and with or without 10 μM FB1 and then incubated at 29 °C. Pictures were taken three days after straining. See Materials and Methods for details.
