# Supplementary material for: Sphingolipid Long-Chain Base Signaling in Compatible and Non-Compatible Plant–Pathogen Interactions in Arabidopsis
Source: Int J Mol Sci. 2023 Feb 23;24(5):4384. doi: 10.3390/ijms24054384 (PMC10002605; doi:10.3390/ijms24054384)
Supplement: Supplementary file 1 [file ijms-24-04384-s001.zip › Supplemental Figure S2.pdf]

Supplemental Figure S2

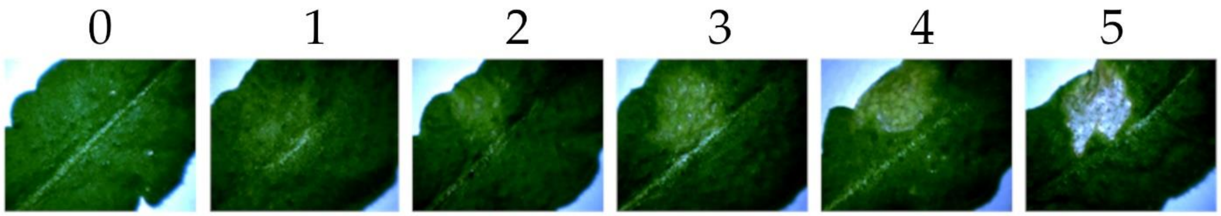

Supplemental Figure S2. Scale to calculate lesion severity (LS) of Fumonisin B1 infiltrated leaves. This was based on representative disease lesions produced by 10 μM FB1 infiltration on leaves from *Arabidopsis thaliana* Col-0 adult plants. For Figure 1d, the progress of the lesion induced by FB1 was assessed at 4 d post-infiltration. The severity of the damage was determined by estimation according to the illustrated scale, where n =0 (no damage) to n=5 (severe damage). The lesion severity (LS) was calculated from these values according to the equation:

$LF = \Sigma$ (leaves $\times n$)/total number of leaves.

See Material and Methods for details.
