# Supplementary material for: Sphingolipid Long-Chain Base Signaling in Compatible and Non-Compatible Plant–Pathogen Interactions in Arabidopsis
Source: Int J Mol Sci. 2023 Feb 23;24(5):4384. doi: 10.3390/ijms24054384 (PMC10002605; doi:10.3390/ijms24054384)
Supplement: Supplementary file 1 [file ijms-24-04384-s001.zip › Supplemental Table S1.pdf]

Supplemental Table S1. *Arabidopsis thaliana* null mutant lines used in this study. The identification numbers, nature of the gene modification, description of their behaviour upon treatments (control, FB1 or avirulent pathogen) and content of the LCBs are described for every genotype.

Mutant line	Gene name		Description	Endogenous content of LCBs nmol g dw $^{-1}$ Sphinganine (SN, d18:0)	Endogenous content of LCBs nmol g dw $^{-1}$ Phytosphingosine (PS, t18:0)
lcb2a- 1 SALK_061472	*LCB2a* (At5g23670)	- FB1	LCB2a has functional redundancy with LCB2b. Although both genes are ubiquitously expressed, *AtLCB2a* expression is higher than that of *AtLCB2b* in most organs of the plant. Under normal growth conditions this mutant has similar LCB content to wild type, but also shows a reduced activity of serine palmitoyltransferase (SPT) (Dietrich et al. 2008).	14.52 ± 1.62*	2605.4 ± 279.44*
				14.82 ± 5.01**	2012.01 ± 166.16**
		+ FB1 72 h	T-DNA insertion in *LCB2a* gene enhances tolerance to FB1 (Saucedo-García et al. 2011).	1122.01 ± 203.79*	31951.43 ± 2261.79*
				350.34 ± 75.86**	12828.69 ± 1123.90**
sbh1-1 SALK_090881	*SBH1-1* (At1g69640)	- FB1	The mutant lacks the activity of one of the hydroxylases (SBH1) that converts the SN sphingoid base to PS, (Chen et al. 2008).	0.99 ± 0.06*	1.75 ± 0.58*
				4.29 ± 0.95**	1.02 ± 0.18**
		+ FB1	The sensitivity of the mutant *sbh1-1* is similar to that of wild-type plants (Saucedo-García et al. 2011).	Not reported	Not reported
		avrRPM1 2 h	After infiltration with the avirulent strain (avrRPM1), wild-type plants accumulate 15-fold PS as compared to SN. The mutant *sbh1-1* is unable to reach this increase but instead shows an upregulation of SN upon infection (Peer et al., 2018).	1.58 ± 0.01*	24.18 ± 5.71*
				10.49 ± 1.41**	1.36 ± 0.01**
mpk6 SALK_073907	*MPK6* At2g43790	- FB1	*mpk6* plants have a dramatic reduction in male fertility. Do not cause alteration in seeds morphology, but the embryo and mature seed are bigger than wild-type (Liu and Zhang; 2004, Müller et al. 2015).	Not reported	Not reported
		+ FB1	The *mpk6* seedlings are less susceptible to FB1 than wild type (Saucedo-García et al. 2011).	Not reported	Not reported

*Wild type
** Mutant

References

Chen, M.; Markham, J.E.; Dietrich, C.R.; Jaworski, J.G.; Cahoon, E.B. Sphingolipid long-chain base hydroxylation is important for growth and regulation of sphingolipid content and composition in Arabidopsis. *Plant Cell*. **2008**, *20*, 1862-1878. doi: 10.1105/tpc.107.057851.

Dietrich, C.R.; Han, G.; Chen, M.; Berg, R.H.; Dunn, T.M.; Cahoon, E.B. Loss-of-function mutations and inducible RNAi suppression of Arabidopsis LCB2 genes reveal the critical role of sphingolipids in gametophytic and sporophytic cell viability. *Plant J.* **2008**, *54*, 284-98. doi: 10.1111/j.1365-313X.2008.03420.x.
Liu, Y.; Zhang, S. Phosphorylation of 1-aminocyclopropane-1-carboxylic acid synthase by MPK6, a stress-responsive mitogen-activated protein kinase, induces ethylene biosynthesis in Arabidopsis. *Plant Cell.* **2004,** *16*, 3386-99. doi: 10.1105/tpc.104.026609.
Müller, J.; Beck, M.; Mettbach, U.; Komis, G.; Hause, G.; Menzel, D.; Samaj, J. Arabidopsis MPK6 is involved in cell division plane control during early root development, and localizes to the pre-prophase band, phragmoplast, trans-Golgi network and plasma membrane. *Plant J.* **2010**, 61, 234-48. doi: 10.1111/j.1365-313X.2009.04046.x.
