# Supplementary material for: Sphingolipid Long-Chain Base Signaling in Compatible and Non-Compatible Plant–Pathogen Interactions in Arabidopsis
Source: Int J Mol Sci. 2023 Feb 23;24(5):4384. doi: 10.3390/ijms24054384 (PMC10002605; doi:10.3390/ijms24054384)
Supplement: Supplementary file 1 [file ijms-24-04384-s001.zip › Supplemental Table S2.pdf]

Supplemental Table S2. Inhibition of production of NADPH oxidase-derived superoxide by DPI in protoplasts stimulated with FB1. Protoplasts were exposed to the indicated concentrations of DPI and FB1 and XTT reduction was measured after 1 h, as described under Material and Methods. The results show the average of three independent experiments.

DPI (μM)	FB1 (μM)	Superoxide production (% control)	Inhibition of O_2^- by DPI (%)
0	0	100	
0	10	292.75	
50	10	238.04	18.49
