# Supplementary material for: Sphingolipid Long-Chain Base Signaling in Compatible and Non-Compatible Plant–Pathogen Interactions in Arabidopsis
Source: Int J Mol Sci. 2023 Feb 23;24(5):4384. doi: 10.3390/ijms24054384 (PMC10002605; doi:10.3390/ijms24054384)
Supplement: Supplementary file 1 [file ijms-24-04384-s001.zip › Supplemental Table S3.pdf]

Supplemental Table S3. Inhibition of production of NADPH oxidase-derived superoxide by DPI in protoplasts stimulated with *Pseudomonas syringae* DC3000 (*avrRPM1*). Protoplasts were exposed to *Pst* DC3000 (avrRPM1) at a concentration of $1x10^7$ CFU/ml and superoxide formation was measured by XTT reduction after 1 h, as described under Material and Methods. DPI was used at the indicated concentrations The results show the average of three independent experiments.

**DPI (μM)**	**Pst DC3000 (*avrRPM1*) (CFU/ml)**	**Superoxide production (% control)**	**Inhibition of O_2^- by DPI (%)**
0	0	100	
0	10^7	305.5	
50	10^7	130.00	57.4
